# Ranking novel cancer driving synthetic lethal gene pairs using TCGA data

**DOI:** 10.18632/oncotarget.10536

**Published:** 2016-07-11

**Authors:** Hao Ye, Xiuhua Zhang, Yunqin Chen, Qi Liu, Jia Wei

**Affiliations:** ^1^ R&D Information, AstraZeneca, Shanghai, China; ^2^ AEM iMed, AstraZeneca, Shanghai, China; ^3^ Department of Central Laboratory, Shanghai Tenth People's Hospital, School of Life Sciences and Technology, Tongji University, Shanghai, China

**Keywords:** synthetic lethality, semi-supervised ranking model, siRNA validation

## Abstract

Synthetic lethality (SL) has emerged as a promising approach to cancer therapy. In contrast to the costly and labour-intensive genome-wide siRNA or CRISPR-based human cell line screening approaches, computational approaches to prioritize potential synthetic lethality pairs for further experimental validation represent an attractive alternative. In this study, we propose an efficient and comprehensive in-silico pipeline to rank novel SL gene pairs by mining vast amounts of accumulated tumor high-throughput sequencing data in *The Cancer Genome Atlas* (TCGA), coupled with other protein interaction networks and cell line information. Our pipeline integrates three significant features, including mutation coverage in *TCGA*, driver mutation probability and the quantified cancer network information centrality, into a ranking model for SL gene pair identification, which is presented as the first learning-based method for SL identification. As a result, 107 potential SL gene pairs were obtained from the top 10 results covering 11 cancers. Functional analysis of these genes indicated that several promising pathways were identified, including the DNA repair related Fanconi Anemia pathway and HIF-1 signaling pathway. In addition, 4 SL pairs, mTOR-TP53, VEGFR2-TP53, EGFR-TP53, ATM-PRKCA, were validated using drug sensitivity information in the cancer cell line databases *CCLE* or *NCI60*. Interestingly, significant differences in the cell growth of mTOR siRNA or EGFR siRNA knock-down were detected between cancer cells with wild type TP53 and mutant TP53. Our study indicates that the pre-screening of potential SL gene pairs based on the large genomics data repertoire of tumor tissues and cancer cell lines could substantially expedite the identification of synthetic lethal gene pairs for cancer therapy.

## INTRODUCTION

Synthetic lethality describes the genetic interaction by which the combination of two separately non-lethal mutations results in lethality. The phenomenon was first described by Calvin Bridges in 1922 [1], who noticed that some combinations of mutations in the model organism *Drosophila melanogaster* confer lethality. Generally, the ablation of two genes located in parallel pathways (leading to cell survival or a common essential product) is one of the important patterns causing synthetic lethality (SL) [2].

Cancer is fundamentally a genetic disease with numerous gene mutations involved. Some of these genetic mutations serve as biomarkers in cancers. In particular, notable advances have been made in cancer therapy for example, with the discovery of Herceptin to treat breast cancer patients with HER2 amplification, and with Iressa for the treatment of non-small cell lung cancer patients with an EGFR mutation. However, developing drugs that selectively kill cancer cells without harming normal cells remains a big challenge in oncology therapy. Given that genetic mutations underpin differences between cancer cells and healthy cells, Hartwell [3] was the first to suggest the use of chemical and genetic synthetic lethality screening for cancer therapy. Since then, this approach has attracted great attention from cancer biologists as it provides a promising perspective for oncology medicine discovery [4, 5]. For example, targeting the PARP-1 enzyme using Olaparib in ovarian cancer patients carrying a tumor BRCA1/2 mutation achieved milestone success in this area [6]. Ultimately, siRNA and CRISPR screenings are the most reliable methods for detecting SL gene pairs. However, compared to model genetic systems (such as yeast or fruit flies), human cell systems hold greater challenges for genome-wide siRNA or CRISPR screening. For this reason, several computational approaches have been proposed to facilitate the systematic detection of SL gene pairs in cancer. Briefly, these methods can be divided into three categories according to their targeted data resources: (i) inferring human ortholog gene pairs from yeast SL genes [7]; (ii) using the robustness features to evaluate the importance of gene pairs in the cancer PPI network [8]; (iii) calculating mutual exclusivity using statistical models from gene mutation/transcriptional expression data [9–12]. More recently, Livnat et al. [13] proposed *DAISY* to identify SL gene pairs. This approach combines somatic copy number alteration, siRNA screening as well as cell survival and gene co-expression information. Derived from comprehensive in-house data, this approach achieved a promising performance in data-driving SL gene pair identification. Nevertheless, we comprehensively compared the four available predicted SL data sets from previously developed methods (including *DAISY*) [8, 10, 12, 13] on SL gene pair prediction. The concordance of predicted SL gene pairs among those different methods is extremely low (see details in *Discussions*). This inconsistency across different methods may indicate that the *in silico* SL gene pair identification methods are far from mature. In addition, none of the previous methods was learning-based, that is, SL gene pair identification was based on the screening of certain criteria rather than training and prediction. We noticed that a portion of known SL gene pairs have been accumulated, and the investigation of the characteristics of these SL gene pairs are expected to derive significant features which can quantitatively depict the common mechanisms of the SL. Therefore, in our study, we designed a learning-based pipeline to rank novel SL gene pairs based on the known SL gene pairs, together with other unknown ones. By mining the accumulated *TCGA* mutation and gene expression data, as well as the gene properties in the protein-protein interaction network, our pipeline can be treated as an integration of the traditional strategies, and ranked a list of potential SL gene pairs. In contrast to the lack of experimental validation in most previous methods, we implemented further siRNA knock-down experiments to evaluate our results.

## RESULTS

### Brief results of 10 times 5-fold cross validation

**Figure 1:**
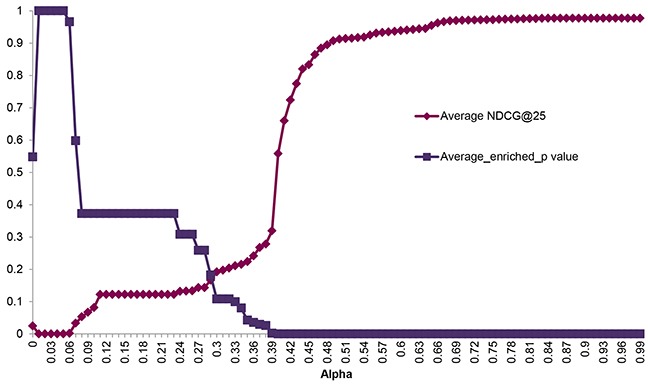
Ranking performance according to alpha The *X*-axis indicates the parameter alpha in manifold ranking algorithm. The *Y*-axis represents the corresponding enriched *p* value and *NDCG* in the top 25 ranking results.

**Figure 2:**
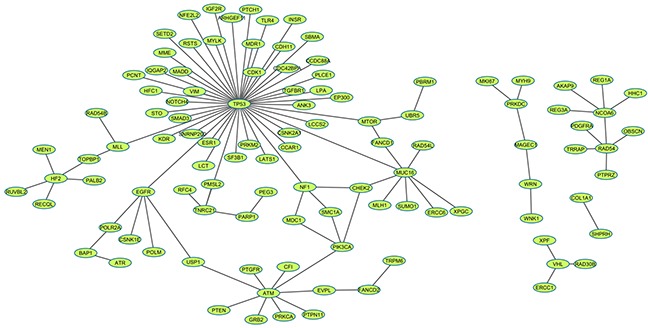
SL network of the predicted 107 pairs Each node represents a gene. The SL relationship of a gene pair was linked by an edge. The graph was generated by *Cytoscape*.

We evaluated the ranking performance in 11 cancers through 10 times 5-fold cross validation, the other cancers in TCGA failed due to the limited number of overlapping samples between mutation data and the expression data or limited coverage of positive SL pairs. The brief results were listed in Table 1. Herein we didn't intend to describe the details of each pair, Kidney renal clear cell carcinoma (KIRC) would be picked out as an example for illustration (see details in Supplementary Table S1). Currently, TCGA mutation data contains 417 KIRC patients. In addition, the gene-expression data on 400 KIRC patients is available in the TCGA dataset. At first, 528 genes with mutation rate >= 1% were selected from the gene mutation data. Following the workflow described in *methods*, 1014 candidate SL gene pairs were generated with the chi-square test *p* value <=0.05 and mutation exclusivity ≥0.8. Three features (Gene pair mutation coverage, Driver mutation probability, Network information centrality) were subsequently calculated. The same calculation process was used on the 119 positive SL gene pairs covered by KIRC mutation and expression data and the cancer network. During the 10 times 5-fold cross validation procedure, the test set contained 23 or 24 SL pairs. Then the top 25 results were used for NDCG calculation and enrichment evaluation, respectively. *alpha* optimization is a critical process in data manifolds ranking algorithm, which can directly influence the ranking performance. As it was shown in Figure 1, NDCG@25 was gradually increased from 0 to 0.9768, as the increase of *alpha*; while the enrichment *p* value was decreased. This means that the ranking performance was better at a larger *alpha*. Finally, the optimized *alpha* = 0.84 was achieved when NDCG@25 reached the peak value. Then, after all of the positive SL pairs were imported as the training set with the optimized *alpha*, we generated a ranking list for the 1014 candidate pairs according to their relevance to positive pairs. The same process was implemented on 10 other cancer types. Finally, we generated a SL network in Figure 2, which is comprised of 107 predicted SL gene pairs from the top 10 results in the 11 cancer types. (See all of the ranking results in Supplementary Table S2)

**Table 1:** Ranking performance in 11 cancer types

Cancer Type	Candidate pair Num.	Positive pair Num.	NDCG@Positive pair/5	Enriched p value	Optimized alpha
LGG	1192	76	0.9808	5.96E-13	0.56
KIRC	1014	119	0.9768	3.90E-13	0.84
CESC	93	38	0.9343	0.0027	0.98
OV	126	99	0.5574	1.50E-07	0.86
BRCA	534	148	0.4618	0.0099	0.99
GBM	791	49	0.3417	0.0262	0.99
LUAD	766	164	0.3244	0.0609	0.99
LUSC	2177	101	0.2928	5.29E-03	0.99
SKCM	7290	135	0.2707	6.24E-04	0.98
HNSC	2213	140	0.2643	0.0271	0.87
STAD	1417	135	0.2372	0.0160	0.98

### Function analysis of the genes in the predicted novel SL pairs

Pathway enrichment was utilized to decipher the biological functions of the gene list. Specifically, 73/107 genes were mapped to *KEGG* pathways. From Table 2, we can see that these genes are mainly enriched in 15 pathways (covering 61.64% of the mapped genes) involved in six biological process categories, namely, replication and repair, signal transduction, the endocrine system, cell growth and death, cellular immunity and development. In particular, the Fanconi Anemia pathway ranked No.1 as a potential pathway for identifying novel anticancer therapies by exploiting synthetic lethal relationships [14]. The most famous example of this is the synthetic lethality relationship between the BRCA1/2 gene in the Fanconi Anemia pathways and PARP [15]. Recently, the first-in-class PARP inhibitor, Olaparib, was approved by the U.S. FDA for use in advanced ovarian cancer patients with BRCA mutations [16]. Based on our findings, several other biological pathways for synthetic lethality exploration were identified. For example, the HIF-1 signalling pathway (which activates the transcription of genes involved in angiogenesis, cell survival, glucose metabolism and invasion), was used as a screening resource in discovering synthetic lethal gene pairs [17]. RAS signalling [18], P53 signalling [19], PI3K-AKT signalling [20], are also widely considered to be promising pathways for synthetic lethal pair identification, and have previously attracted considerable research interest.

**Table 2:** Enriched pathways of the top 10 results in 11 cancer types

Pathway Category	Pathway	Predicted SL genes in the pathway	Number of the predicted SL genes	p value
Replication and repair	Fanconi anemia pathway	ERCC1; PMS2; USP1; PALB2; ATR; FANCD2; BRCA2; ERCC4; MLH1; POLI	10	3.06E-08
Signal transduction	FoxO signaling pathway	ATM; TGFBR1; PIK3CA; PTEN; INSR; CREBBP; EP300; SMAD3; GRB2; EGFR; MAPK1; CSNK1E	12	7.02E-07
Signal transduction	HIF-1 signaling pathway	VHL; PIK3CA; INSR; CREBBP; MTOR; EP300; EGFR; TLR4; PRKCA; MAPK1	10	8.57E-06
Endocrine system	Thyroid hormone signaling pathway	TP53; PIK3CA; CREBBP; MTOR; EP300; NOTCH4; ESR1; PLCE1; PRKCA; MAPK1	10	1.55E-05
Cell growth and death	Cell cycle	TP53; ATM; CDK1; PRKDC; CREBBP; CHEK2; EP300; ATR; SMAD3; SMC1A	10	2.68E-05
Cellular community	Adherens junction	TGFBR1; INSR; CREBBP; EP300; SMAD3; EGFR; CSNK2A1; MAPK1	8	3.60E-05
Signal transduction	Ras signaling pathway	PDGFRA; PIK3CA; INSR; PTPN11; GRB2; EGFR; NF1; KDR; PLCE1; PRKCA; MAPK1	11	0.00039
Cellular community	Focal adhesion	PDGFRA; PIK3CA; PTEN; COL1A1; MYLK; GRB2; EGFR; KDR; PRKCA; MAPK1	10	0.00067
Signal transduction	ErbB signaling pathway	PIK3CA; ERBB3; MTOR; GRB2; EGFR; PRKCA; MAPK1	7	0.00071
Signal transduction	PI3K-AKT signaling pathway	PDGFRA; TP53; PIK3CA; PTEN; COL1A1; INSR; MTOR; GRB2; EGFR; KDR; TLR4; PRKCA; MAPK1	13	0.00087
Cell growth and death	p53 signaling pathway	TP53; ATM; CDK1; PTEN; CHEK2; ATR	6	0.0016
Replication and repair	Nucleotide excision repair	ERCC1; ERCC5; ERCC4; RFC4; ERCC6	5	0.0031
Development	Dorso-ventral axis formation	GRB2; EGFR; NOTCH4; MAPK1	4	0.0041
Cellular community	Gap junction	PDGFRA; CDK1; GRB2; EGFR; PRKCA; MAPK1	6	0.0049
Signal transduction	Wnt signaling pathway	TP53; CREBBP; EP300; SMAD3; CSNK2A1; PRKCA; CSNK1E	7	0.0098

### *In-vitro* drug sensitivity of cell lines in which one of the genes in each synthetic lethality pair is targeted

In the process of validating the SL gene pairs in the cell lines, two strict criteria are applied in the cell response information for each SL gene pair: 1. One of the genes in a SL pair should be the target of a drug in the database. 2. Several cancer cell lines treated by the drug must possess the mutation of the other gene in the SL pair. Finally, only 37 predicted SL gene pairs could be well annotated by the drug sensitivity data in *CCLE* [21] and *NCI60* [22] databases (see Table 3 and Table 4). We compared the drug sensitivity of the two types of cancer cell lines, namely, the cell lines with the other gene mutation and the cell lines without the other gene mutation in the SL pair. We found 4 pairs: mTOR-TP53, VEGFR2-TP53, EGFR-TP53, ATM-PRKCA which showed significantly higher drug sensitivity (targeting one of the genes in the SL pairs) in the cell lines with the other gene mutations (*P* value <= 0.05), than the cell lines without the other gene mutation in these predicted SL pairs.

**Table 3:** Comparison of drug sensitivity between two groups of cell lines in CCLE data

Pair(Entrz Gene ID)	Gene A	Gene B	Drug targeted on gene A	Mean of drug sensitivity (PIC50)^§^		*p* value
				Cell lines with gene B mutation	Cell lines with gene B wild type	
1009_7157	TP53	CDH11	Nutlin-3	5.0978	5.1246	0.7689
11200_4763	CHEK2	NF1	AZD7762	6.1902	6.0711	0.1553
11200_5290	PIK3CA	CHEK2	GDC0941	5.1880	5.1031	0.3676
	PIK3CA	CHEK2	NVP-BEZ235	6.9181	6.9876	0.6393
	CHEK2	PIK3CA	AZD7762	5.8728	6.1251	0.9818
1387_7157	TP53	RSTS	Nutlin-3	5.1032	5.1261	0.8182
1956_1454	EGFR	CSNK1E	Gefitinib	5.1213	4.9910	0.2948
	EGFR	CSNK1E	Lapatinib	5.1555	5.0623	0.3428
	EGFR	CSNK1E	BIBW2992	5.1208	5.0253	0.3775
	EGFR	CSNK1E	Erlotinib	5.0803	5.0415	0.4258
	EGFR	CSNK1E	ZD-6474	5.1210	5.2915	0.8464
1956_7157	EGFR	TP53	BIBW2992	5.1074	4.8862	**0.0047**
	EGFR	TP53	Gefitinib	5.0485	4.8969	**0.0121**
	EGFR	TP53	ZD-6474	5.3078	5.2584	0.0679
	EGFR	TP53	Erlotinib	5.0587	5.0137	0.1822
	EGFR	TP53	Lapatinib	5.0810	5.0339	0.1933
	TP53	EGFR	Nutlin-3	5.1081	5.1247	0.7205
2065_7157	TP53	LCCS2	Nutlin-3	5.1057	5.1251	0.7572
2475_51366	MTOR	UBR5	Temsirolimus	6.4909	6.4765	0.4590
2475_7157	TP53	MTOR	Nutlin-3	5.1135	5.1240	0.6411
	MTOR	TP53	Temsirolimus	6.4561	6.5175	0.7357
3643_7157	TP53	INSR	Nutlin-3	5.1204	5.1232	0.5272
367_7157	TP53	SBMA	Nutlin-3	5.0970	5.1248	0.7850
3791_7157	TP53	VEGFR2	Nutlin-3	5.1186	5.1234	0.5590
	VEGFR2	TP53	Sorafenib	5.0474	5.1525	0.9738
4297_7157	TP53	MLL	Nutlin-3	5.1239	5.1230	0.4885
4638_7157	TP53	MYLK	Nutlin-3	5.1091	5.1320	0.9036
472_2885	ATM	GRB2	KU-55933	3.6089	3.8219	0.8999
472_3426	ATM	CFI	KU-55933	3.7372	3.8174	0.6780
472_5290	PIK3CA	ATM	GDC0941	5.1010	5.1066	0.5171
	ATM	PIK3CA	KU-55933	3.7713	3.8224	0.7299
	PIK3CA	ATM	NVP-BEZ235	6.9180	6.9958	0.7795
472_5578	ATM	PRKCA	KU-55933	3.9089	3.7880	**0.0423**
472_5728	ATM	PTEN	KU-55933	3.8008	3.8179	0.5858
472_5781	ATM	PTPN11	KU-55933	3.6390	3.8214	0.8728
4763_7157	TP53	NF1	Nutlin-3	5.1033	5.1260	0.8145
4855_7157	TP53	NOTCH4	Nutlin-3	5.1386	5.1184	0.1609
546_5156	PDGFRA	RAD54	Pazopanib	3.8402	4.1592	0.9844
	PDGFRA	RAD54	Sorafenib	4.9588	5.1008	0.9495
5727_7157	TP53	PTCH1	Nutlin-3	5.1016	5.1248	0.7610
64324_7157	TP53	STO	Nutlin-3	5.1031	5.1244	0.7307
2475_675	MTOR	BRCA2	Temsirolimus	6.3225	6.4942	0.8553
7099_7157	TP53	TLR4	Nutlin-3	5.1121	5.1236	0.6151
7157_1457	TP53	CSNK2A1	Nutlin-3	5.0969	5.1236	0.6783
7157_2033	TP53	EP300	Nutlin-3	5.1252	5.1219	0.4262
7157_2099	TP53	ESR1	Nutlin-3	5.0986	5.1238	0.6970
7157_4088	TP53	SMAD3	Nutlin-3	5.1190	5.1231	0.5239
7157_5594	TP53	PRKM2	Nutlin-3	5.0969	5.1233	0.6144
7157_7046	TP53	TGFBR1	Nutlin-3	5.0969	5.1240	0.7224
7157_983	TP53	CDK1	Nutlin-3	5.0969	5.1233	0.6276
	CDK1	TP53	RO-3306	4.0215	4.0474	0.6447
8476_7157	TP53	CDC42BPA	Nutlin-3	5.1285	5.1226	0.4244
9113_7157	TP53	LATS1	Nutlin-3	5.0969	5.1247	0.7767

§ PIC50 means negative log_10_(IC50) values (higher value indicate higher drug sensitivity)

**Table 4:** Comparison of drug sensitivity between two groups of cell lines in NCI60 data

Pair (Entrz Gene ID)	GeneA	Gene B	Drug targeted on gene A	Mean of drug sensitivity (z-score normalized GI50 values)*		*p* value
				Cell lines with gene B mutation	Cell lines with gene B wild type	
1956_7157	EGFR	TP53	Lapatinib	−0.1547	0.2300	0.0791
	EGFR	TP53	Erlotinib hydrochloride	−0.0997	0.0925	0.2517
	EGFR	TP53	Gefitinib	−0.0414	0.1119	0.2968
	EGFR	TP53	Afatinib	−0.0551	−0.0219	0.4541
2475_7157	MTOR	TP53	Sirolimus	−0.2149	0.3950	**0.0070**
	MTOR	TP53	Temsirolimus	−0.1846	0.4443	**0.0146**
	MTOR	TP53	Everolimus	−0.1158	0.2619	0.0771
367_7157	SBMA	TP53	Dromostanolone Propionate	−0.1572	0.2575	0.0519
	SBMA	TP53	Calusterone	−0.0156	0.1200	0.3373
	SBMA	TP53	Nandrolone phenpropionate	0.0200	−0.0169	0.5508
3791_7157	VEGFR2	TP53	Pazopanib hydrochloride	−0.2302	0.4594	**0.0052**
	VEGFR2	TP53	Axitinib	−0.1028	0.2731	0.1062
	VEGFR2	TP53	Sunitinib malate/Sunitinib (free base)	−0.0886	0.17125	0.1696
7157_2099	ESR1	TP53	Raloxifene hydrochloride	−0.1808	0.2231	0.0740
	ESR1	TP53	Fulvestrant	−0.1194	0.2606	0.1093
	ESR1	TP53	Tamoxifen citrate	−0.0158	0.0525	0.3947
	ESR1	TP53	Estramustine phosphate sodium	−0.1011	−0.0450	0.4061
7157_5594	PRKM2	TP53	Arsenic Trioxide	−0.0272	0.0275	0.4218

* z score normalized GI50 values are the elements of cellular fingerprint in NCI60 dataset. (http://data-analysis.charite.de/care/index.php?site=about#usecase) (Smaller values indicate higher drug sensitivity)

### Possible molecular mechanisms of the 4 positive pairs

According to Kaelin [23]'s synthetic lethality model, synthetic lethality occurs via 4 different mechanisms: The cellular organizational units may be uniquely redundant and their roles are essential (type A), subunits of an essential multi-protein complex (type B), interconnected components in an essential linear pathway (type C), or they may participate in parallel pathways that are together essential (type D). The 4 pairs were consistent with either type D or type C.

### mTOR-TP53

mTOR can integrate nutrient and mitogen signals to activate cell growth (increase cell mass and cell size) and cell division [24, 25], whilst one of the most important functions of TP53 is its ability to activate apoptosis [26]. Cell growth and apoptosis may provide parallel functions in cancer pathology. mTOR and TP53 may be considered synthetic lethality targets.

### VEGFR2-TP53, EGFR-TP53

EGFR is a hot target for cancer therapy with many currently FDA approved drugs, and can activate at least 4 major downstream signalling cascades including; RAS-RAF-MEK-ERK, PI3 kinase-AKT, PLCgamma-PKC and STATs modules. Those signalling cascades can ultimately lead to a series of cellular events such as cell proliferation, inhibition of apoptosis, angiogenesis, migration, adhesion and invasion [27]. Furthermore, VEGF's can specifically induce blood and lymphatic vessel development and homeostasis [28]. Inhibition of EGFR can block the angiogenesis process. Double knock out VEGFR2 and TP53 may lead to synthetic lethality through angiogenesis and apoptosis.

### ATM-PRKCA

ATM [29] is a key regulator of multiple signaling cascades that respond to DNA damage. These responses involve the activation of cell cycle checkpoint factors, DNA repair and apoptosis. PRKCA has long been recognized to participate in activating tumour growth and development across different cancers [30]. In addition, PRKCA activation can result in increased cell motility in several *in vivo* and *in vitro* cancer models, the effect of which may be reversed with PRKCA inhibition [31, 32]. Hence, ATM and PRKCA knock-out, coupled with loss of function of apoptosis and the cell migration process, may generate synthetic lethality.

### Validation through siRNA knock-down in cancer cell lines

**Figure 3:**
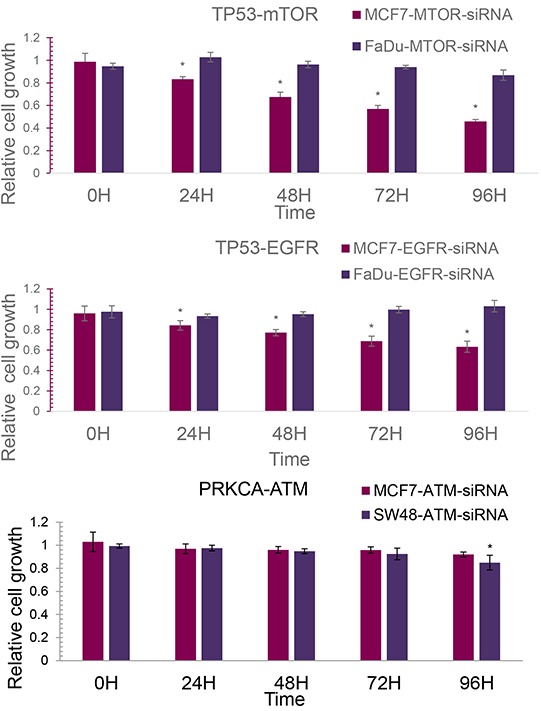
The relative cell growth of cancer cell lines **A.** The relative cell growth of MCF7 and FaDu with siRNA knock-down of mTOR. **B.** The relative cell growth of MCF7 and FaDu with siRNA knock-down of EGFR. **C.** The relative cell growth of MCF7 and SW48 with siRNA knock-down of ATM.

Regarding the mutation information in the *CancerDR* database [33], three cancer cell lines were selected (Table 5). MCF-7 is a breast cancer cell line, carrying wild type TP53 and PRKCA. FaDu is a human epithelial cell line with mutant TP53 and SW48 is an invasive human colon adenocarcinoma cell line with a PRKCA mutation. Since an extremely low level of VEGFR2 mRNA expression was detected in the FaDu line, only three gene pairs (TP53-mTOR, TP53-EGFR, PRKCA-ATM) were able to be analyzed for siRNA knock-down validation. The relative cell growth results are displayed in Figure 3.

**Table 5:** siRNA knock-down on the cancer cell lines

SL pair (Entrz Gene ID)	Gene A	Cell line with wild type gene A	Cell line with mutant gene A	siRNA knock down gene B
7157-2475	TP53	MCF-7	FaDu	MTOR
7157-1956	TP53	MCF-7	FaDu	EGFR
7157-3791	TP53	MCF-7	FaDu	VEGFR2
472-5578	PRKCA	MCF-7	SW48	ATM

### siRNA knock-down validation on TP53-mTOR

According to the results of mRNA expression detection, there was no significant difference in the mTOR knock-down rate between MCF7 and FaDu (average mTOR knock-down rate: MCF7 62.26%; FaDu 60.67%). As shown in Figure 3A, the cell growth inhibitory effect of mTOR knock-down was observed in both MCF7 and FaDu. 96 hours after mTOR siRNA knock-down, the relative cell growth of FaDu slightly decreased from 1.1596 to 0.8178, while the relative cell growth of MCF7 dramatically decreased from 0.9509 to 0.4581. 24 hours after mTOR knock-down, the relative cell growth of MCF7 was always significantly lower than FaDu with a t-test *P* value of less than 0.01. The lowest inhibition of cell growth (54.19%) was achieved after 96 hours of mTOR knockdown in the MCF7 cells. This may indicate that wild type TP53 in the MCF7 line can possibly strongly enhance the cell growth inhibition effects of mTOR knock-down, compared to the mutant TP53 in FaDu.

### siRNA knock-down validation on TP53-EGFR

Also, no significant difference in the EGFR siRNA knock-down rate was detected between MCF7 and FaDu. The optimal knock-down rates were 82.81% and 88.87% in FaDu and MCF7, respectively. Figure 3B displays the relative cell growth of MCF7 and FaDu cells after EGFR siRNA knock-down within 96 hours. No inhibition of cell growth using EGFR knock-down was observed in FaDu cells. The average relative cell growth was maintained at around 0.9325 ~ 1.0300 upon EGFR knock-down, whilst the relative cell growth of MCF7 strongly decreased from 0.9595 to 0.6884 following EGFR knock-down. 24 hours after EGFR knock-down, the relative cell growth of the MCF7 line was always significantly lower than the FaDu line with a t-test *P* value of less than 0.01. The wild type TP53 in MCF7 cells with EGFR knock-down could lead to cell growth inhibition.

### siRNA knock-down validation on PRKCA-ATM

Upon siRNA transfection, the average knock-down rates of ATM were 86.07% and 45.43% in MCF7 and SW48 cells, respectively. Figure 3C shows the relative cell growth of MCF7 and SW48 cells with ATM siRNA knock-down. The relative cell growth of MCF cells decreased slightly from 1.030 to 0.9208. Considering the high ATM siRNA knock-down efficiency, this suggests that ATM knock-down has very limited inhibition effects on MCF cell growth. For SW48 cells, the relative cell growth decreased from 0.9936 to 0.8495. 96 hours after ATM knock-down, the relative cell growth of SW48 was significantly lower than MCF7 (0.8495 ± 0.0209 vs. 0.9208 ±0.0636, t test *P* value = 0.01296). This may partly indicate that the mutant PRKCA in SW48 cells can enhance the inhibition of cell growth on ATM knock-down, compared to the wild type PRKCA in MCF7. The slight inhibition observed on SW48 cell growth may be caused by the low ATM siRNA knock-down rate in the cell. It may be a novel SL gene pair with further rigorous validation.

## DISCUSSION

Regarding the predicted SL gene pairs: mTOR-TP53 and EGFR-TP53, we found that mTOR knock-down or EGFR knock-down could cause much stronger cell growth inhibition in cell lines with wild type TP53, compared to mutant TP53. It seems that the relationships between mTOR-TP53 and EGFR-TP53 are exactly opposed to the concept of synthetic lethality. Indeed, the multifunctional nature of mutant TP53 needs to be better understood. A series of earlier studies [34–37] had suggested that mutant TP53 not only represents the equivalent of wild type TP53 functional loss, but also acquires new functions in driving cell migration, invasion and metastasis. The significant differences in cell growth between cancer cell lines with wild type TP53 and mutant TP53 could partly suggest that there is a special relationship between TP53-mTOR and TP53-EGFR. Notably, TP53 may be a promising biomarker in the development of cancer drugs targeting the mTOR and EGFR pathways in precision medicine.

siRNA knock-down is limited by both the expression level of the gene in the cell and the knock-down efficiency of the siRNA. In our study, we failed to validate VEGFR2-TP53 due to the low expression levels of VEGFR2 in the FaDu cell line. The low knock-down rate (45%) of ATM may weaken the inhibitory effects on cell growth. In response to these issues, the latest CRISPR technology [38, 39], which can provide considerable gene editing power, may provide a more reliable approach to further validate SL gene pairs.

**Figure 4:**
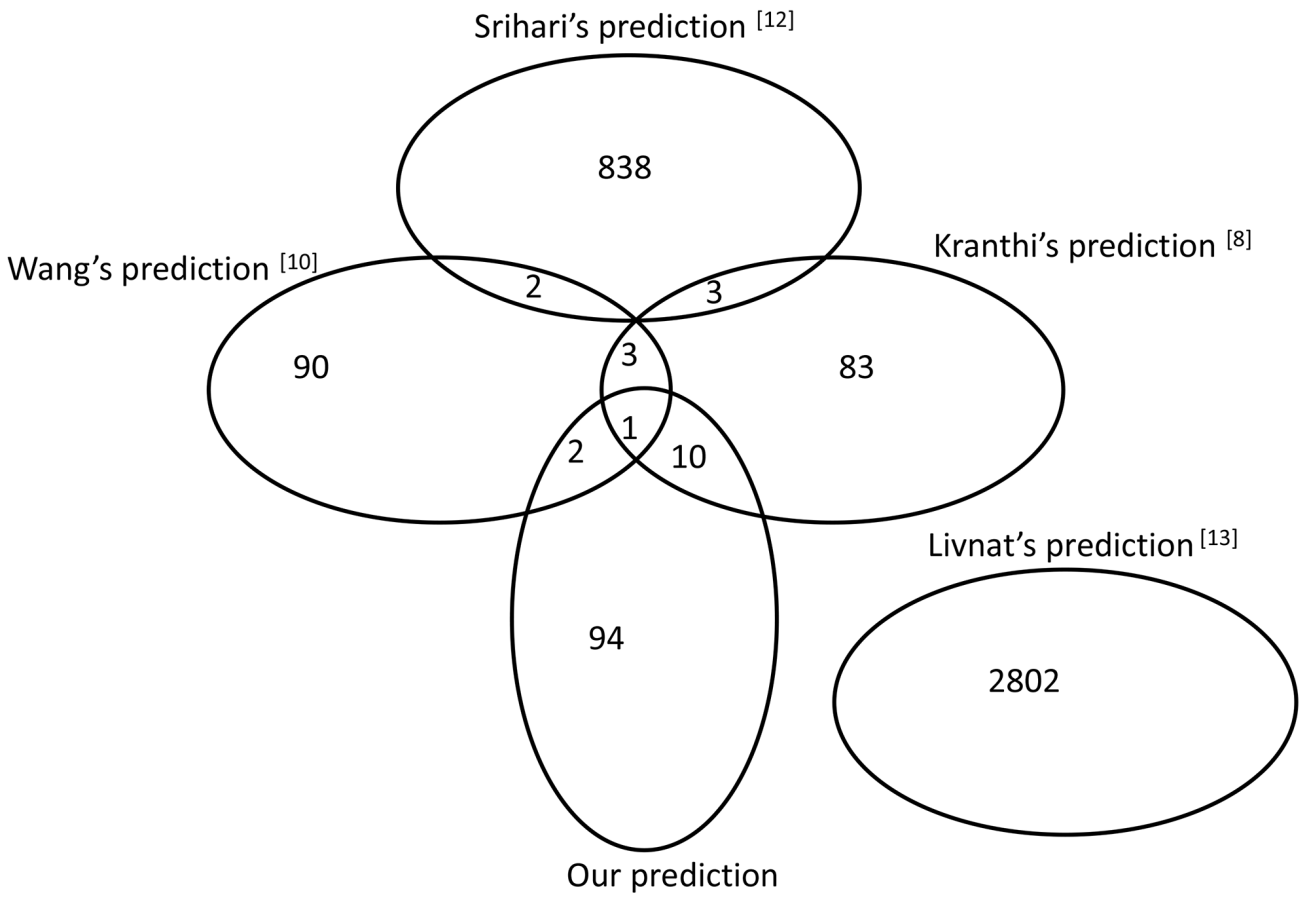
Comparison of the predicted results with other methods The Venn diagram was drawn based on the overlap of the predicted SL gene pairs in three previous reports and our results.

Detecting SL gene pairs in humans is a challenging problem due to the highly evolved, complex and redundant signalling pathways within human cells. The influence of a loss of function caused by gene mutation can often be complemented by parallel pathway signalling. Various computational methods can provide potential SL gene pairs from different perspectives, such as the correlation of gene expression with mutation, robustness in the cancer network or gene co-expression in related biological processes. In this study, we compared the 107 predicted SL pairs with the results of four previous methods (see details in Supplementary Table S3). As shown in Figure 4, 12.15% (13 pairs) of our predicted results overlapped with Wang's [10] or Kranthi's [8] prediction. Importantly, TP53-mTOR and EGFR-TP53 pairs validated by drug sensitivity data were included in the overlapping pairs. This may suggest that overlapping predictions from different methods may provide more reliable results. Interestingly, we also found that no overlap occurred between Livnat's [13] predictions or any of the other four methods. The original input data may be one of the important factors in influencing the final predictions. Kranthi's method [8] started with the human protein-protein interaction database HPRD [40] as well as CancerGenes [22]. Wang's prediction [10] was based on the profiling data of glioblastoma multiforme from *TCGA* as well as the p53 mutation information from the *Trust Sanger Institute*. Srihari [12] used the copy-number and gene-expression profiling data of four cancers (breast, prostate, ovarian and uterine) in *TCGA* as input data for their method. The different features of these input data across the methods may generate bias in SL gene pair predictions. Since there was no *priori* knowledge of cancer targets in the NCI-60 database, *CancerGenes* or *Metacore* were used to filter the input data in Livnat's model [13], the potential SL gene pairs from the cancer lines may lead to distinct prediction results from others. In addition, due to the low concordance of results between different methods, further efforts to explore such complex SL relationships in a human system may be required.

## CONCLUSIONS

In this study, we proposed a semi-supervised ranking pipeline to rank novel SL gene pairs based on the vast amounts of accumulated *TCGA* data. 107 novel potential SL gene pairs were predicted from the top 10 results covering 11 cancers. In particular, 4 SL pairs: mTOR-TP53, VEGFR2-TP53, EGFR-TP53, ATM-PRKCA, could be validated using drug sensitivity information in the cancer cell line databases *CCLE* or *NCI60*. Furthermore, the results of siRNA knock-down experiments indicated that significant differences in the cell growth of mTOR or EGFR siRNA knock-down were detected between the cancer cells with wild type TP53 and mutant TP53. The TP53 mutation may serve as a biomarker for cancer therapy in drugs targeting mTOR or EGFR. More promisingly, a recent study [41] has proposed P53 as a biomarker for predicting the progression free survival (PFS) of pancreatic cancer patients being treated with erlotinib (EGFR inhibitor). Taken together, these data underscore the potential of investigating the role of P53 as a predictive biomarker in other cancer types.

## MATERIALS AND METHODS

### SL gene pair prediction pipeline

In this study, we designed a semi-supervised learning model [42] to rank the similarities between positive SL gene pairs and candidate SL gene pairs, mainly using 3 defined features namely, gene pair mutation coverage, driver mutation probability and the quantified network information centrality. More specifically, we used three features to describe both the known SL gene pairs and candidate SL gene pairs. Then the semi-supervised method could rank the candidate SL gene pairs according to the similarity of these features with the known SL gene pairs. Herein, gene pair mutation coverage was defined as the percentage of samples containing at least one gene mutation in the pair. Furthermore, in order to get more reliable results from TCGA mutation data, the mutations of genes in candidate SL pairs should be covered by a certain number of samples. Driver mutations play vital roles in cancer development. Regarding the cancer specific SL pairs, we hypothesised that the mutation of genes playing an important role in cancer progression are more likely to be driver mutations. Last, the network information centrality helps to identify the potential nodes, which are crucial for the proper functioning of the system. Since simultaneously mutating two genes in a SL gene pair could dramatically influence the cellular process and cause cell death, network information centrality was used to calculate the influence of knocking-out a node pair on system stability. This approach inherently mimics the synthetic lethality mechanism well.

**Figure 5:**
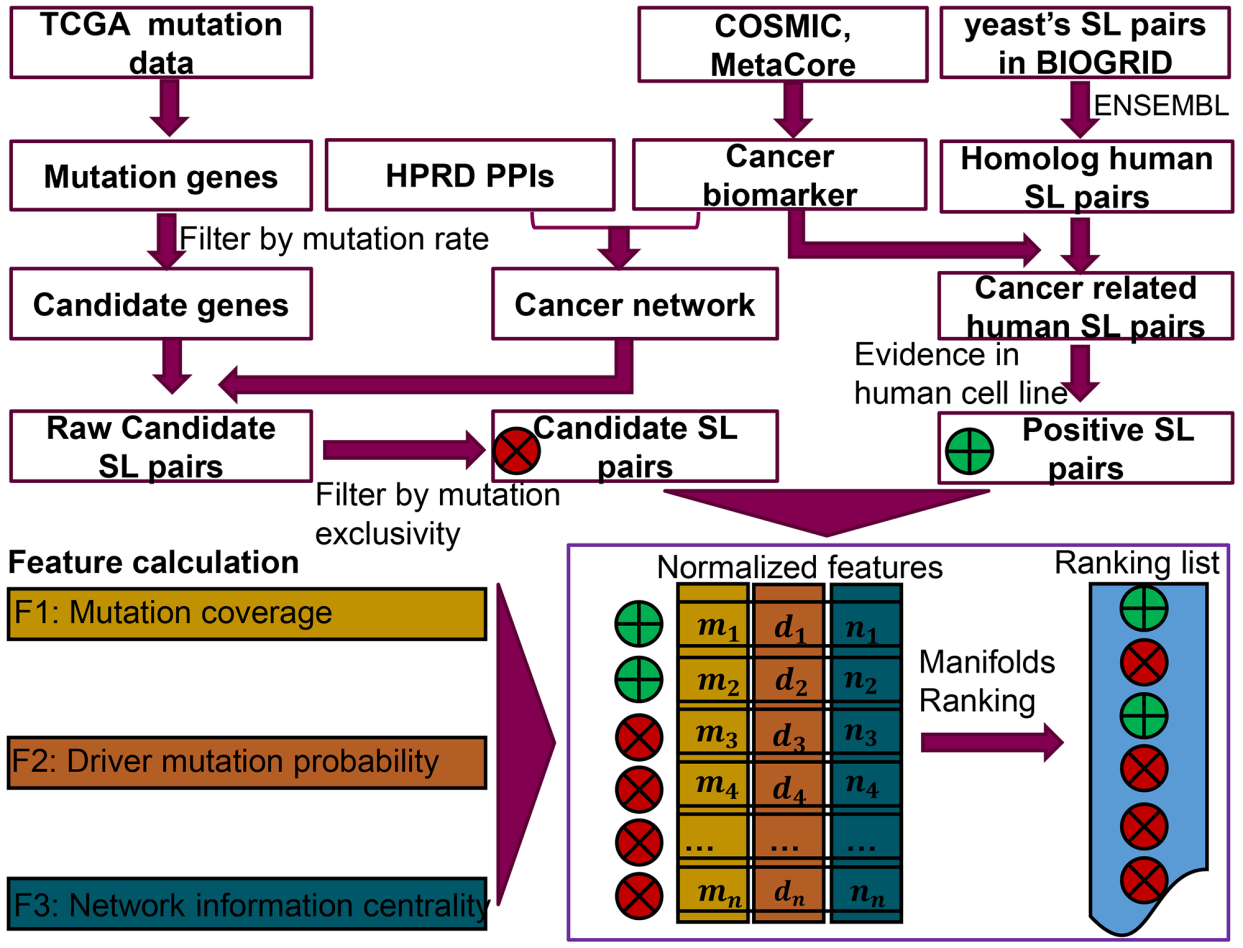
Workflow of the SL prediction pipeline The three types of original data (gene mutation and expression, cancer biomarker, SL-pairs in yeast) were downloaded from *TCGA*, *COSMIC* and *MetaCore* as well as *BIOGRID*, respectively. The cancer network was built from the interaction of cancer biomarkers in the protein-protein interaction database *HPRD*. Each raw candidate SL pair was composed of a highly mutated gene and another gene in the cancer network. Then a chi-square test on the mutation exclusivity with *p* value <= 0.05 was utilized to generate the candidate SL pairs, while the positive SL pairs were derived from the yeasts’ SL pairs followed by homology transformation, cancer biomarker filtration and literature evidence identification on human cell lines. For each pair in of candidate SL and positive SL data, three features were generated for them. The first feature was calculated from the mutation coverage of each SL gene pair in the *TCGA* mutation data. The driver mutation probability was calculated by *R* package *DriverNet*. The third feature was defined to evaluate the influence on stability of the cancer network, after removing the two genes from an SL pair. Then normalized features of each SL pair were imported into a manifolds ranking model to generate a ranking list of potential SL pairs.

The brief workflow of the SL prediction pipeline is shown in Figure 5. In the first step, cancer biomarkers were collected from *COSMIC* [43] and *MetaCore* [44], which were used as a filter to select raw cancer related SL pairs. Next, the positive SL pairs were generated from yeast SL pairs, followed by homolog gene transformation, cancer biomarker filtering as well as the application of evidence in human cell lines obtained from literature mining. The candidate genes were selected from *TCGA* mutation data. The raw candidate SL pairs were then composed based on a candidate gene and a gene within a cancer network. Then, a Chi-square test (implemented by *chi2_contingency* in python package *Scipy*) was used to evaluate whether the mutations of the two genes is an independent event in each raw candidate SL pair. In addition, the mutation exclusivity was also calculated, which was defined as the percentage of samples carrying one of the mutant genes in the SL gene pair [9]. Only those independent gene mutations with high mutation exclusivity were selected as candidate SL pairs for further calculation. Subsequently, three features of both candidate SL pairs and positive SL pairs were calculated and normalized before being exported into a learning model. Finally, the novel SL pairs were detected with an optimized parameter which was obtained from 10 times 5-fold cross validation.

### *TCGA* mutation and expression data processing

We downloaded *TCGA* mutation and expression profiling data from the UCSC Cancer Genomics Browser (https://genome-cancer.ucsc.edu), which provides well-annotated and interactive visualizations of *TCGA* genomic, phenotypic, and clinical data [45]. We then obtained two matrices. Each row of the matrices represented a gene, and each column indicated a sample. The values in the cells represented the expression and mutation status in the gene expression matrix and the gene mutation matrix, respectively. Finally, data from 11 cancers, containing both the gene expression matrix and the corresponding gene mutation matrix, were used in our study.

### Positive synthetic lethality gene pairs

The collective data on yeast SL (synthetic lethal) genes based on high throughput genetic screening is available at *BioGRID* [46]. However, no curated database of human SL gene pairs has been established yet. In this study, *BioGRID* was used as the primary resource to retrieve human cancer related SL gene pairs. The phylogenetic inference from yeast to human genes was obtained from the Ensemble database (http://useast.ensembl.org/). Then, homolog human SL pairs were filtered by cancer biomarkers in *MetaCore* (https://portal.genego.com/) and driver genes in *COSMIC* [43]. Only homolog human SL gene pairs with both of the genes covered by cancer biomarkers or driver genes were kept for downstream analysis. In order to reduce the false positive rate as much as possible, for each homolog human SL gene pair, we checked the evidence available in the PubMed literature. Finally, 399 positive SL pairs were identified with the evidence of synthetic lethality in human cell lines or animal models in the literature (see Supplementary Table S4).

### Cancer network

307,066 protein-protein interactions were downloaded from *HPRD* [40]. Then, we used cancer biomarkers from *MetaCore* and *COSMIC* [43] to filter them. In details, we searched the keywords ‘cancer, tumor, carcinoma’ in *MetaCore* and retrieved 4,296 cancer related biomarkers. At the same time, we also downloaded the 507 driver mutation genes collected in the Cancer Gene Census from the website of *COSMIC*. All of these gene mutations in Cancer Gene Census have been proved to causally implicate in cancer. Then, for each protein-protein interaction, only if both proteins are included in *MetaCore* cancer biomarkers or Cancer Gene Census in *COSMIC*, would the protein-protein interaction be kept. Finally, we obtained 11,925 protein-protein interaction pairs, corresponding to 2,869 individual proteins. The cancer network could be built with edge presented by the protein-protein interaction, as well as the node displayed by a protein.

### Candidate SL pairs generation

We calculated the mutation rate of each gene among the samples in the *TCGA* mutation data. Herein, 1% was utilized as the cut-off threshold to select the candidate genes. Each raw candidate SL gene pair was generated by selecting a candidate gene as well as the other gene from the cancer network. Subsequently, we tested whether *gene A* mutation and *gene B* mutation are independent events based on the mutation data. In detail, the null hypothesis is that *gene A* mutation and *gene B* mutation are independent of each other. A Chi-square test was implemented on a 2×2 contingency table (see Table 6). *M* represents the number of samples carrying both *gene A* and *gene B* mutations; *N* represents the number of samples carrying the *gene A* mutation without the *gene B* mutation; *X* represents the number of samples carrying the *gene B* mutation without the *gene A* mutation; *Y* is the number of samples that containing both wild type *gene A* and wild type *gene B*.

**Table 6:** 2×2 contingency table in chi-square test

	*gene B* +	*gene B* −
*gene A* +	*M*	*N*
*gene A* −	*X*	*Y*

+ mutant type

− wild type

The raw candidate SL gene pairs with Chi-square test p value <=0.05 means the mutation of *gene A* and *gene B* are not independent. Maybe some relationships exist between mutation of *gene A* and *gene B.* In addition, the mutation exclusivity of *gene A* and *gene B* could be calculated as (X+N)/(M+N+X). The higher mutation exclusivity indicates the gene A and gene B are more likely to be mutually exclusive mutations. Herein, only candidate SL pairs with both Chi-square test P value ≤ 0.05 and mutation exclusivity ≥ 0.8 were selected for the downstream processing.

### Features calculation

#### Gene pair mutation coverage

It was defined as the percentage of samples containing at least one gene mutation in the pair. For example, *gene A* is mutated in samples *s1,s3,s6, gene B* is mutated in samples *s3,s8,s9*. *n* is the total number of samples. The mutation coverage of the pair (*gene A, gene B*) is 5/*n*.

#### Driver mutation probability

Herein, we utilized the *R* package *DriverNet* [47] to evaluate the driver mutation probability of genes based on the relationship between mutation and consequent changes in gene expression. The input data of *DriverNet* comes from two matrices, namely a mutation matrix and its corresponding gene expression matrix. Each column of the two matrices is a sample, whilst each row represents the mutation status or expression level of a gene among the samples. The output of *DriverNet* is the *P* value of each gene that will likely be a driver of gene mutation. The smaller *P* value of the two genes from a SL pair was transformed to a negative log10 (*P* value) indicating the strength of the driver mutation for the pair.

#### Network information centrality

If *G* refers to the cancer network mentioned above, and *G’* refers to the cancer network after removing *gene A* and *gene B*, then the network information centrality of *gene A* and *gene B* could be defined as formula I:

$$C_{\text{geneA},\text{geneB}}=\left|\frac{\Delta E}{E}\right|=\left|\frac{E\left(G\right)-E\left(G^{\prime }\right)}{E\left(G\right)}\right|$$ (I)

Where *E*(*G*) is the efficiency of the network. It could be calculated in the formula II:

$$E\left(G\right)=\frac{1}{N\left(N-1\right)}\Sigma_{i\neq j;i,j\;G}\frac{1}{d_{\text{ij}}}$$ (II)

Herein, if *gene i* could reach *gene j* in cancer network, *d*_*ij*_ is the length of the shortest path between the *gene i* and *gene j* (calculated by *shortest_path_length* in python package *networkx*)*,* otherwise, *d*_*ij*_ is equal to *D(G) +* 1*. D(G)* represents the diameter of cancer network, which is defined as the largest distance across all of the shortest paths in the cancer network (calculated through *diameter* in python package *networkx*).

Finally, normalization of the three features was taken to transform the values of each feature between 0-1 in formula III. *x* is the original value of a feature. *x’* is the normalized value.

$$x^{\prime }=\frac{x-\min\left(x\right)}{\max\left(x\right)-\min\left(x\right)}$$ (III)

### A semi-supervised ranking model

The principle of our ranking model, which is referred to as a manifold ranking algorithm [42, 48] can be intuitively explained: the problem is defined in two datasets, a true sample set and an unknown sample set (background); and the goal is to rank the individual members of the unknown sample set according to their *relevance* to the true samples. This model is well suited to address our problem scenario, which is that we only have few known SL pairs in hand (known positive data samples), and we want to prioritize the largest possible gene pair combinations based on their possibility to be the true SL pairs. In detail, we used three features to describe each SL pair. Then 1- cosine angle distance was calculated to represent the *relevance* between candidate SL pairs and true SL pairs.

**Input**: A set of points *X* = (*x_1_*… *x_q_*, *x_q + 1_*… *x_n_*) representing the SL pairs. The first *q* points are true SL pairs, while, the others are candidate SL pairs. The initial score *y* was defined as (1…1,0 …0). (The true SL pairs are corresponding to 1, candidate SL pairs are assigned as 0.) Define *f^0^* = *y*; α is a parameter of the algorithm.

**Output**: A ranked list of *X*, where higher ranked gene pairs are more likely to be SL gene pairs.

1. Define the similarity matrix *W_ij_* = 1- *cosine(i,j)* and *W_ii_* = 0.
2. Compute *L* = *D^−1/2^WD^−1/2^* with *D* being a diagonal matrix defined as $D_{\text{ii}}=\sum_{j=1}^{n}W_{\text{ij}}$
3. Set iteratively *f^t+1^* = *αLf^t^* + *(1-α)y* until f converges, where α is a parameter in [0, 1);
4. Let f* be the converged function *f^t^*; and rank all the points *X* in the decreasing order of their f* values.

It has been shown [42] that *f* * could be calculated as formula IV.

$$f^{*}=\left(1-\alpha \right){\left(I-\alpha L\right)}^{\left(-1\right)}y$$ (IV)

### Evaluation test design

#### 10 times 5-fold cross validation

For each case, the positive SL pairs were divided into five segments. Four of them were used as training sets, while the rest of the segments were used for evaluation. Next, positive SL pairs were shuffled 10 times, the overall performance was determined by the average results of these 10 shuffling events.

#### Ranking performance evaluation

Normalized discounted cumulative gain (*NDCG*) [49] was originally used to evaluate web search engine algorithms in the field of information retrieval. It can measure the usefulness of a document based on its position in the result list. Here we used NDCG to measure the effectiveness of ranking performance for each case's predicted results (see formula V).

$$\text{NDCG}@p=Z•\sum_{i=1}^{p}\frac{2^{{\text{rel}}_{i}}-1}{\log_{2}\left(i+1\right)}$$ (V)

*Z* is the normalization constant.

*i* is the rank position of candidate SL pair *m*.

*rel_i_* is the relevance value of candidate SL pair *m*. If candidate SL pair *m* belongs to the positive SL pairs, *rel_i_* is set to 1, otherwise, *rel_i_* is set to 0.

*p* is the maximum position.

For example, if the three positive SL pairs are ranked at 2, 3 & 8, respectively, while the ideal rank position should be 1, 2 & 3, then:

$$Z=\frac{1}{\sum_{i=1}^{3}\frac{1}{\log_{2}\left(i+1\right)}}=0.4331$$

In the top 5 results,

$$\text{NDCG}@5=Z•\left(\frac{1}{\log_{2}\left(2+1\right)}+\frac{1}{\log_{2}\left(3+1\right)}\right)=0.4907$$

In addition, the positive enrichment of SL pairs in the top *n* ranking position are also used to evaluate our prediction performance. Herein, a hypergeometric test is utilized. (see formula VI)

$$p=1-\sum_{x=0}^{k}\frac{C_{M}^{x}•C_{N-M}^{n-x}}{C_{N}^{n}}$$ (VI)

*k*: number of positive SL pairs included in top *n* ranking results.

*N*: the whole candidate SL pairs

*M*: the whole positive SL pairs.

### Comparison of drug sensitivities between two groups of cancer cell lines

The original drug sensitivity data, drug targets as well as the mutation backgrounds of cancer cell lines in *CCLE* [21], *NCI60* [22] were downloaded from broadinstitute.org/ccle/home and discover.nci.nih.gov/cellminer/, respectively. Regarding a SL gene pair, if one of the genes in the SL pair is targeted by a drug, we compared the drug sensitivities on the cell lines carrying the mutation of the other genes in the pair and the cell lines containing the wild type of the other genes. The lower GI50 or IC50 value means higher drug sensitivity.

### Further validation through siRNA knock down on cell lines

In order to get more reliable validation of the predicted SL gene pairs, we conducted siRNA knock-down experiments on cancer cell lines. The influence on cell growth of different genetic background cell lines would indicate SL relationships.

For example, regarding a SL gene pair *gene a* –*gene b*, two cancer cell lines were selected. The first cell line carried mutant *gene a*, while the wild type *gene a* was carried in the other cell line. Then, the siRNA of *gene b* was transfected into the two cell lines. We recorded the cell growth of two cell lines at the time points of 0h, 24h, 48h, 72h and 96h on two different treatments: placebo, siRNA-knockdown of *gene b*, respectively. Herein, we did 8 parallel experiments at each time point. The relative cell growth was calculated through the formula VII.

$$\text{Relative cell growth}=\frac{\text{Growth of cell with siRNA treatment}}{\text{Growth of cell with placebo treatment}}$$ (VII)

## SUPPLEMENTARY TABLES











## References

[1] Nijman SM (2011). Synthetic lethality: general principles, utility and detection using genetic screens in human cells. FEBS Lett.

[2] Canaani D (2014). Application of the concept synthetic lethality toward anticancer therapy: A promise fulfilled?. Cancer letters.

[3] Hartwell LH, Szankasi P, Roberts CJ, Murray AW, Friend SH (1997). Integrating genetic approaches into the discovery of anticancer drugs. Science.

[4] Kaelin WG (2009). Synthetic lethality: a framework for the development of wiser cancer therapeutics. Genome Med.

[5] Luo J, Solimini NL, Elledge SJ (2009). Principles of cancer therapy: oncogene and non-oncogene addiction. Cell.

[6] Eskander RN, Tewari KS (2014). PARP inhibition and synthetic lethality in ovarian cancer. Expert Rev Clin Pharmacol.

[7] Conde-Pueyo N, Munteanu A, Sole RV, Rodriguez-Caso C (2009). Human synthetic lethal inference as potential anti-cancer target gene detection. BMC Syst Biol.

[8] Kranthi T, Rao SB, Manimaran P (2013). Identification of synthetic lethal pairs in biological systems through network information centrality. Mol Biosyst.

[9] Miller CA, Settle SH, Sulman EP, Aldape KD, Milosavljevic A (2011). Discovering functional modules by identifying recurrent and mutually exclusive mutational patterns in tumors. BMC Med Genomics.

[10] Wang X, Simon R (2013). Identification of potential synthetic lethal genes to p53 using a computational biology approach. BMC Med Genomics.

[11] Ciriello G, Cerami E, Sander C, Schultz N (2012). Mutual exclusivity analysis identifies oncogenic network modules. Genome Res.

[12] Srihari S, Singla J, Wong L, Ragan MA (2015). Inferring synthetic lethal interactions from mutual exclusivity of genetic events in cancer. Biology direct.

[13] Jerby-Arnon L, Pfetzer N, Waldman YY, McGarry L, James D, Shanks E, Seashore-Ludlow B, Weinstock A, Geiger T, Clemons PA (2014). Predicting cancer-specific vulnerability via data-driven detection of synthetic lethality. Cell.

[14] Jenkins C, Kan J, Hoatlin ME (2012). Targeting the Fanconi Anemia Pathway to Identify Tailored Anticancer Therapeutics. Anemia.

[15] Fong PC, Boss DS, Yap TA, Tutt A, Wu P, Mergui-Roelvink M, Mortimer P, Swaisland H, Lau A, O'Connor MJ (2009). Inhibition of poly (ADP-ribose) polymerase in tumors from BRCA mutation carriers. New England Journal of Medicine.

[16] Ray T (2014). FDA Approves First PARP Inhibitor with Myriad's BRACAnalysis as CDx in Advanced Ovarian Cancer. genomeweb: genomeweb).

[17] Jones DT, Harris AL (2012). Small-molecule inhibitors of the HIF pathway and synthetic lethal interactions. Expert Opinion on Therapeutic Targets.

[18] Weidle UH, Maisel D, Eick D (2011). Synthetic Lethality-based Targets for Discovery of New Cancer Therapeutics. Cancer Genomics - Proteomics.

[19] Morandell S, Yaffe MB (2012). Exploiting synthetic lethal interactions between DNA damage signaling, checkpoint control, and p53 for targeted cancer therapy. Prog Mol Biol Transl Sci.

[20] Crowder RJ, Phommaly C, Tao Y, Hoog J, Luo J, Perou CM, Parker JS, Miller MA, Huntsman DG, Lin L (2009). PIK3CA and PIK3CB inhibition produce synthetic lethality when combined with estrogen deprivation in estrogen receptor–positive breast cancer. Cancer research.

[21] Barretina J, Caponigro G, Stransky N, Venkatesan K, Margolin AA, Kim S, Wilson CJ, Lehár J, Kryukov GV, Sonkin D (2012). The Cancer Cell Line Encyclopedia enables predictive modelling of anticancer drug sensitivity. Nature.

[22] Reinhold WC, Sunshine M, Liu H, Varma S, Kohn KW, Morris J, Doroshow J, Pommier Y (2012). CellMiner: a web-based suite of genomic and pharmacologic tools to explore transcript and drug patterns in the NCI-60 cell line set. Cancer research.

[23] Kaelin WG (2005). The concept of synthetic lethality in the context of anticancer therapy. Nature reviews cancer.

[24] Fingar DC, Richardson CJ, Tee AR, Cheatham L, Tsou C, Blenis J (2004). mTOR Controls Cell Cycle Progression through Its Cell Growth Effectors S6K1 and 4E-BP1/Eukaryotic Translation Initiation Factor 4E. Molecular and Cellular Biology.

[25] Laplante M, Sabatini DM (2012). mTOR signaling in growth control and disease. Cell.

[26] Fridman JS, Lowe SW (2003). Control of apoptosis by p53. Oncogene.

[27] Oda K, Matsuoka Y, Funahashi A, Kitano H (2005). A comprehensive pathway map of epidermal growth factor receptor signaling. Molecular systems biology.

[28] Cebe-Suarez S, Zehnder-Fjällman A, Ballmer-Hofer K (2006). The role of VEGF receptors in angiogenesis; complex partnerships. Cellular and molecular life sciences.

[29] Lavin MF, Kozlov S (2007). ATM activation and DNA damage response. Cell cycle.

[30] Kang J-H (2014). Protein Kinase C (PKC) Isozymes and Cancer. New Journal of Science.

[31] Koivunen J, Aaltonen V, Koskela S, Lehenkari P, Laato M, Peltonen J (2004). Protein kinase C α/β inhibitor Go6976 promotes formation of cell junctions and inhibits invasion of urinary bladder carcinoma cells. Cancer research.

[32] Masur K, Lang K, Niggemann B, Zanker KS, Entschladen F (2001). High PKC α and low E-cadherin expression contribute to high migratory activity of colon carcinoma cells. Molecular biology of the cell.

[33] Kumar R, Chaudhary K, Gupta S, Singh H, Kumar S, Gautam A, Kapoor P, Raghava GP (2013). CancerDR: cancer drug resistance database. Scientific reports.

[34] Zhou G, Wang J, Zhao M, Xie TX, Tanaka N, Sano D, Patel AA, Ward AM, Sandulache VC, Jasser SA, Skinner HD, Fitzgerald AL, Osman AA (2014). Gain-of-function mutant p53 promotes cell growth and cancer cell metabolism via inhibition of AMPK activation. Molecular cell.

[35] Oren M, Rotter V (2010). Mutant p53 gain-of-function in cancer. Cold Spring Harbor perspectives in biology.

[36] Dittmer D, Pati S, Zambetti G, Chu S, Teresky AK, Moore M, Finlay C, Levine AJ (1993). Gain of function mutations in p53. Nature genetics.

[37] Muller PA, Vousden KH (2014). Mutant p53 in cancer: new functions and therapeutic opportunities. Cancer cell.

[38] Cong L, Ran FA, Cox D, Lin S, Barretto R, Habib N, Hsu PD, Wu X, Jiang W, Marraffini LA (2013). Multiplex genome engineering using CRISPR/Cas systems. Science.

[39] Doudna JA, Charpentier E (2014). Genome editing. The new frontier of genome engineering with CRISPR-Cas9. Science.

[40] Prasad TK, Goel R, Kandasamy K, Keerthikumar S, Kumar S, Mathivanan S, Telikicherla D, Raju R, Shafreen B, Venugopal A (2009). Human protein reference database—2009 update. Nucleic acids research.

[41] Ormanns S, Siveke JT, Heinemann V, Haas M, Sipos B, Schlitter AM, Esposito I, Jung A, Laubender RP, Kruger S, Vehling-Kaiser U, Winkelmann C, Fischer von Weikersthal L (2014). pERK, pAKT and p53 as tissue biomarkers in erlotinib-treated patients with advanced pancreatic cancer: a translational subgroup analysis from AIO-PK0104. BMC Cancer.

[42] Zhou D, Weston J, Gretton A, Bousquet O (2004). Ranking on data manifolds.

[43] Forbes SA, Bindal N, Bamford S, Cole C, Kok CY, Beare D, Jia M, Shepherd R, Leung K, Menzies A, Teague JW, Campbell PJ, Stratton MR, Futreal PA (2011). COSMIC: mining complete cancer genomes in the Catalogue of Somatic Mutations in Cancer. Nucleic acids research.

[44] Thomson Reuters MetaCore database https://portal.genego.com/.

[45] Cline MS, Craft B, Swatloski T, Goldman M, Ma S, Haussler D, Zhu J (2013). Exploring TCGA Pan-Cancer data at the UCSC Cancer Genomics Browser. Scientific reports.

[46] Chatr-Aryamontri A, Breitkreutz BJ, Heinicke S, Boucher L, Winter A, Stark C, Nixon J, Ramage L, Kolas N, O'Donnell L, Reguly T, Breitkreutz A, Sellam A (2013). The BioGRID interaction database: 2013 update. Nucleic acids research.

[47] Bashashati A, Haffari G, Ding J, Ha G, Lui K, Rosner J, Huntsman DG, Caldas C, Aparicio SA, Shah SP (2012). DriverNet: uncovering the impact of somatic driver mutations on transcriptional networks in cancer. Genome Biol.

[48] Liu Q, Cui J, Yang Q, Xu Y (2010). In-silico prediction of blood-secretory human proteins using a ranking algorithm. BMC bioinformatics.

[49] Järvelin K, Kekäläinen J (2002). Cumulated gain-based evaluation of IR techniques. ACM Transactions on Information Systems (TOIS).

