# Supplementary material for: Ranking novel cancer driving synthetic lethal gene pairs using TCGA data
Source: Oncotarget. 2016 Jul 11;7(34):55352–67. doi: 10.18632/oncotarget.10536 (PMC5342422; doi:10.18632/oncotarget.10536)
Supplement: Supplementary file 1 [file oncotarget-07-55352-s001.pdf]

Ranking novel cancer driving synthetic lethal gene pairs using TCGA data

SUPPLEMENTARY TABLES

Supplementary Table S1: Performance evaluation through 10 times 5-fold cross validation.

See Supplementary Table 1

Supplementary Table S2: SL ranking results.

See Supplementary Table 2

Supplementary Table S3: Comparison on the prediction results of existing methods.

See Supplementary Table 3

Supplementary Table S4: Positive SL gene pair dataset.

See Supplementary Table 4
